# Proliferative and Osteogenic Supportive Effect of VEGF-Loaded Collagen-Chitosan Hydrogel System in Bone Marrow Derived Mesenchymal Stem Cells

**DOI:** 10.3390/pharmaceutics15041297

**Published:** 2023-04-20

**Authors:** Jeevithan Elango

**Affiliations:** 1 Department of Biomaterials Engineering, Faculty of Health Sciences, UCAM-Universidad Católica San Antonio de Murcia, Campus de los Jerónimos 135, Guadalupe, 30107 Murcia, Spain; srijeevithan@gmail.com or jelango@ucam.edu; Tel.: +34-6-0359-7596; 2 Center of Molecular Medicine and Diagnostics (COMManD), Department of Biochemistry, Saveetha Dental College and Hospitals, Saveetha Institute of Medical and Technical Sciences, Saveetha University, Chennai 600077, India; 3 Department of Marine Biopharmacology, College of Food Science and Technology, Shanghai Ocean University, Shanghai 201306, China

**Keywords:** hydrogel, VEGF, collagen–chitosan, mesenchymal stem cells, osteogenesis

## Abstract

The use of hydrogel (HG) in regenerative medicine is an emerging field and thus several approaches have been proposed recently to find an appropriate hydrogel system. In this sense, this study developed a novel HG system using collagen, chitosan, and VEGF composites for culturing mesenchymal stem cells (MSCs), and investigated their ability for osteogenic differentiation and mineral deposition. Our results showed that the HG loaded with 100 ng/mL VEGF (HG-100) significantly supported the proliferation of undifferentiated MSCs, the fibrillary filament structure (HE stain), mineralization (alizarin red S and von Kossa stain), alkaline phosphatase, and the osteogenesis of differentiated MSCs compared to other hydrogels (loaded with 25 and 50 ng/mL VEGF) and control (without hydrogel). HG-100 showed a higher VEGF releasing rate from day 3 to day 7 than other HGs, which substantially supports the proliferative and osteogenic properties of HG-100. However, the HGs did not increase the cell growth in differentiated MSCs on days 14 and 21 due to the confluence state (reach stationary phase) and cell loading ability, regardless of the VEGF content. Similarly, the HGs alone did not stimulate the osteogenesis of MSCs; however, they increased the osteogenic ability of MSCs in presence of osteogenic supplements. Accordingly, a fabricated HG with VEGF could be used as an appropriate system to culture stem cells for bone and dental regeneration.

## 1. Introduction

The research on hydrogels (HG) in drug delivery and tissue regeneration is an emerging field due to its excellent tissue regenerative properties with controllable drug delivery mechanisms. The manipulation of HGs with cells and drugs is fascinating to modern scientists due to their adaptability in seeding any type of biological cells for different purposes such as target drug delivery, drug screening, and therapeutic applications. HGs possess unique features (such as their hydrophilic nature) and provide an excellent microenvironment for various biological cells to facilitate better regenerative abilities. Several studies reported the wound-healing ability of an HG fabricated with cells in the wound site through physical or chemical stimuli. For instance, chitosan-based HGs are well-documented for wound-healing applications with the addition of other biomolecules [1,2,3]. It has been reported that the chitosan HG produced a thermosensitive sol−gel transition by glycerophosphate salt at body temperature [4]. Aside from wound-healing, chitosan-based HGs are also used to grow bone and cartilage cells, which are differentiated from human mesenchymal stem cells (hMSCs) [5,6].

The most common biomaterial used in tissue engineering in addition to chitosan is collagen. Specifically, collagen type I has attracted much attention due to its biomimetic properties being the same as native tissue with excellent biocompatibility and self-regeneration. Therefore, collagen type I has been widely used in fabricating several types of biomaterials such as scaffolds, HGs, bio-inks, films, and 3D matrices for many regenerative applications in tissues as diverse as bone [7], ligaments [8], skin [9] and blood vessels [10]. Type I collagen is a chief structural protein in several tissues and comprises a range of bioactive sites that support cell attachment [11,12] and control cell differentiation [13,14]. The most common binding sites in biological cells are integrins (alpha and beta), DDR, glycoprotein VI, osteoclast-associated receptor (OSCAR), LAIR-1, and uPARAP/Endo180 [15].

Both collagen and chitosan materials are extracted from natural sources and have a huge impact on implantation in regenerative medicine. For instance, collagen–chitosan composites are a well-known system in bone tissue engineering to improve the mechanical properties of scaffolds and also accelerate the osteogenic differentiation of hMSCs [16]. Wang et al., fabricated chitosan/collagen composite materials crosslinked with glyoxal and assessed the osteoconductive and osteogenic potential of composite material in Hbmsc [17]. The drug delivery effect of collagen–chitosan HGs conjugated with the angiopoietin-1-derived peptide was investigated for morphology, viability, and metabolic activity of cardiomyocytes [18]. Recently, Deepthi et al., fabricated a chitosan–collagen HG-coated electrospun membrane of a PLLA nanofiber construct for flexor tendon regeneration [19]. In another study, functionalized single-wall carbon nanotubes were integrated into a collagen–chitosan HG for minimally invasive applications in bone regeneration [20]. Aleem et al., developed a smart HG with collagen–chitosan containing thyroxin in order to stimulate neovascularization [21].

Vascular endothelial growth factor (VEGF) is an important molecule in the regulation of homeostasis of several biological tissues, most specifically in the development of bone, cartilage, dental, and vascular tissues, as well as in angiogenesis [22]. Additionally, VEGF plays a crucial role in maintaining normal physiological processes and several pathologies such as retinopathies and cancer [23,24,25]. Previously, a VEGF-loaded collagen HG promoted the recovery of cerebral ischemia in rats [26], myogenesis and innervation after subcutaneous implantation in nude mice [27], and neural tissue regeneration [28]. Different types of HGs were fabricated with VEGF containing PEGylated fibrinogen and hyaluronic acid for myocardial infarction [29,30], hyaluronic acid for bioprosthetic heart valves [31], decellularized adipose tissue for angiogenesis [32], and fibrinogen-alginate for plasticity in an injured spinal cord [33]. The drug-releasing pattern of VEGF from a collagen HG and its angiogenesis behavior was previously investigated by Tabata et al. [34]. In addition, VEGF was loaded with a collagen HG for neural stem cell regeneration [28], cerebral ischemia in rats [26], pancreatic islet vascularization [35], angiogenesis [36], and bone regeneration [37,38]. In parallel, VEGF was previously incorporated with a chitosan HG for odontogenic dental pulp stem cell differentiation [39,40], therapeutic neovascularization [41], wound healing [42], acute myocardial infarction [43], blood vessel regeneration [44], and bone regeneration [45].

Considering the above hypothesis, regenerative medicine has many challenges regarding the choice of biomaterials that intend to meet the growing needs for therapies for trauma-, disease- or aging-affected bones [46,47,48]. Surprisingly, none of the studies reported the effect of collagen and chitosan HGs fabricated with VEGF for the proliferative, structural, and osteogenic potential of hBMMSCs. Hence, the present study fabricated the collagen–chitosan HGs with different concentrations of VEGF 25, 50, and 100 nM and investigated their ability in hBMMSC proliferation and differentiation.

## 2. Materials and Methods

### 2.1. Materials

Raw materials such as collagen type I and chitosan (85% degree of deacetylation, MW-190 kDa) were purchased from Sigma-Aldrich, St. Louis, MO, USA. HumanKine^®^ recombinant human VEGF165 protein (Reference: HZ-1038-10UG, Primary Activity 0.3–3.75 ng/mL EC50, Purity > 95%, Molecular Mass 45 kDa, Accession Number→P15692) was purchased from Proteintech, Rosemont, IL, USA. All the chemicals and reagents used in this study were procured from local vendors and were analytical grade unless otherwise specified.

### 2.2. Hydrogel Fabrication

**Figure 1:**
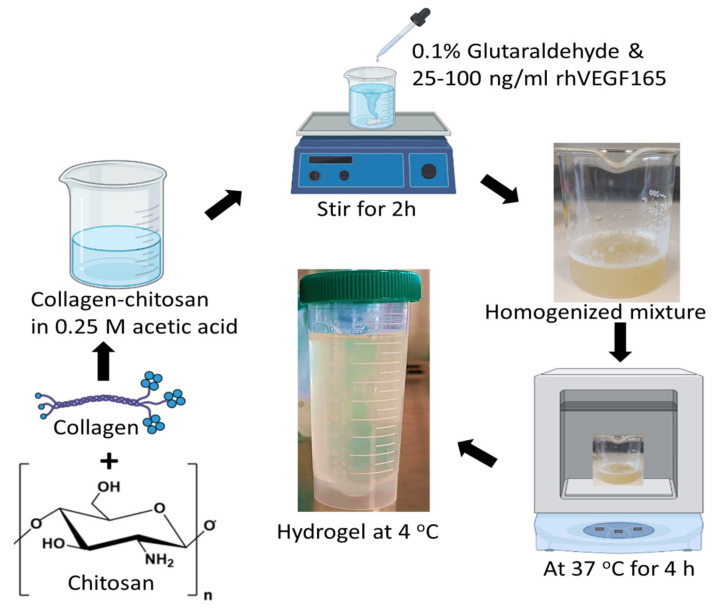
Schematic representation of collagen–chitosan hydrogel fabrication with different contents of VEGF.

Collagen type I and chitosan (85% degree of deacetylation, MW-190 kDa) (Sigma-Aldrich, St. Louis, MO, USA) were used as raw materials for the fabrication of the HG. In brief, the HG was fabricated by mixing 1% collagen and 1% chitosan in 0.25 M acetic acid and stirring for 2 h. Glutaraldehyde with 0.1% to the polymer concentration was used as a cross-linker. After homogenization, the HG (pH~5.0) was incubated at 37 °C for 4 h and then stored at 4 °C for further use. For the VEGF-loaded HG, 25, 50, and 100 ng/mL VEGF (*v:v*, ratio) were added separately into the HG mixture [36,39,40] and followed the same protocol as above (Figure 1). HGs loaded with 25, 50, and 100 ng/mL VEGF were labeled as HG-25, HG-50, and HG-100, respectively.

### 2.3. In Vitro Release Profile of VEGF

The release pattern of VEGF over different incubation durations was determined by using the ELISA method. Briefly, the HGs (~1 mL) were incubated in PBS (1 mL) for 3 h, 6 h, 1 d, 2 d, 3 d, 4 d, 5 d, 6 d, and 7 d at 37 °C. In each time point, 100 μL of PBS from each HG was used for the release profile using an ELISA kit as per the manufacturer’s instruction (Cat No. EH0327, Wuhan Fine Biotech Co., Ltd., Wuhan, China), and the final release profile of VEGF was calculated for 1 mL.

### 2.4. In Vitro Cell Culture

In vitro cell culturing was carried out using human bone mesenchymal stem cells (hMSCs). hMSCs were purchased from LGC Standards, Barcelona, Spain (ATCC PCS-500-012, LGC Standards, Barcelona, Spain, Order Ref. No. 86605340). The primary culture of hMSCs (5 × 10^5^ cells/vial) was performed as per the guidelines of the supplier. Briefly, the cells were cultured in a mesenchymal specific medium (PCS-500-030) supplemented with 5% fetal bovine serum (FBS) (Gibco, Waltham, MA, USA), 1% antibiotic (penicillin- streptomycin-Amphotericin B) (PCS-999-002), and mesenchymal stem cell growth factors (15 ng/mL rh IGF-1, 125 pg/mL Rh FGF-b, 2.4 mM L-Alanyl-L-Glutamine (PCS-500-041) for 5–7 days with medium change twice a week. The cells were sub-cultured once they reached 80% confluence. The monolayer of cells was trypsinized using 0.25% Trypsin with EDTA and spun at 200 g for 5 min. The cell pellet was resuspended in an MSC culture medium and used for the following experiments. The cells from passages 3–7 were used for all the experiments.

### 2.5. Proliferation

The proliferation of MSCs cultured on the HG was carried out by following our previous protocol [49]. Briefly, the cells were cultured in a T75 cm^2^ culture flask until reaching 80% confluence, then were harvested for proliferation assay. Then, the cultured cells were seeded with a cell density of 5 × 10^4^ per well in 24-well cell culture plates. Before seeding the cells, the HG was sterilized under UV for 12 h. For the proliferation assay, the cells seeded on different HGs in 24-well culture plates were cultured in a CO_2_ incubator with a relative humidity of 95% and 5% CO_2_ for 1, 3, and 7 days. The number of cells proliferated with and without HGs was measured as per our previous method [49] by using an automated cell counter (Invitrogen, Countess 3 FL, Thermo Fisher Scientific, Waltham, MA, USA). The cells cultured without HGs were considered the control.

### 2.6. Cytotoxicity

For cytotoxic assessment, the cells were seeded on the HG as described in Section 2.3. The cytotoxic effect of HG was determined by using the MTT kit at 1, 3, and 7 days. Briefly, 50 μL MTT of reagent (5 mg/1 mL PBS) was added into each well at the respective time and incubated for 4 h in a CO_2_ incubator. Then, the unreacted MTT reagent was washed out using PBS wash and 200 μL of DMSO was added into each well in order to solubilize the formazan crystal. The amount of formed formazan crystal was quantified spectrometrically at 570 nm by taking 100 μL from each well using a SpectraMax iD3 Multi-Mode Microplate Reader. The cells cultured without HG were considered the control.

### 2.7. Cell Seeding Density

The total capacity of HGs in cell seeding was determined by seeding the cells (2 × 10^5^) on the HG for 5 h. Before cell seeding, the 48 well culture plates were completely coated with the HG (0.5 mL) for 2 h at 37 °C (Supplementary Figure S1) and the number of bound cells on the HG was quantified by transferring the HG with cells into new wells and the cell number was quantified by trypsinization with 0.25% trypsin-EDTA and an Invitrogen automated cell counter. The cells seeded without an HG were considered the control.

### 2.8. Cell Morphological Properties

In order to investigate the cell morphological properties cultured on an HG, the H&E staining method was used. Briefly, the cells cultured on an HG for 1, 3, and 7 days were washed with PBS, fixed in formalin for 30 min, hematoxylin for 2 min, bluing reagent for 1 min, dehydration, eosin for 2 min, and finally underwent gradient dehydration (0, 25%, 50%, 75%, and 100% ethanol). The images were captured by using a bright-field microscope. The cells cultured without an HG were considered the control.

### 2.9. Osteogenesis (Proliferation)

The efficiency of the HG in osteogenesis was investigated as per our previous method [49]. Briefly, the cells with a cell density of 5 × 10^5^ were seeded on HGs in 12-well culture plates and were cultured with an osteogenic culture medium and supplements (Gibco, Reference: A10066-01, Lot No: 2419081) for 14 and 21 days. The culture medium was replaced every two days until the end of the experiments. The cells cultured without an HG and osteogenic medium (MSC medium) were considered the control and negative control, respectively. The proliferative rate of differentiated MSC cells cultured on the HG was determined on days 14 and 21 as mentioned before using an Invitrogen automated cell counter.

### 2.10. Mineral Stain

To determine the osteogenic potential of HGs, the amount of mineral formed (mineralization) in differentiated osteogenic cells was visualized using alizarin red and von Kossa stain. To do this, the cells were seeded on collagen–chitosan–VEGF HG-coated 12-well plates with a cell density of 5 × 10^5^ per well and were cultured for 14 and 21 days. The medium was replaced every two days until the experiment ended. At each point, the culture medium was removed and the cells were washed gently with PBS and fixed with 4% paraformaldehyde (PFA) for 30 min as per our previous method [49]. Then, the cells were stained with alizarin red stain (3%) and von Kossa stain (LabClinics, Barcelona, Spain) for 30 min under dark conditions, and the unbound excess stains were removed by a PBS wash. Alizarin red and von Kossa stain were used to stain the deposited calcium red and calcium phosphate black/brown in differentiated MSCs, respectively. The amount of alizarin and von Kossa staining was quantified as per our previous method [49]. Briefly, the quantification of staining in differentiated cells was carried out by using the online tool ImageJ software (Version 1.52n), and the staining amount of the test group was compared with the control group (without hydrogel) by considering the control to be 100%. 

### 2.11. Alkaline Phosphatase (ALP) Stain

We further investigated the osteogenic stimulatory effect of a collagen–chitosan HG loaded with VEGF. To do this, a well-known hallmark osteogenic marker, ALP, was used by following our previous protocol. Briefly, the cells with a density of 5 × 10^5^ were seeded on 12-well cell culture plates previously coated with a collagen–chitosan HG loaded with VEGF and were cultured for 14 and 21 days. The medium was changed twice a week until the end of the experiments. At each time point, the culture medium was removed and the cells were washed with PBS followed by histological fixation using 2.5% glutaraldehyde and 4% paraformaldehyde for 30 min each. Then the cells were briefly washed again with PBS and stained with an ALP kit (Cat No. SCR004, Sigma-Aldrich, Madrid, Spain) as recommended by the manufacturer.

### 2.12. Statistical Analysis

The data were represented as mean and standard deviation and the level of statistical significance was determined by using GraphPrism 9.0.1 (GraphPad Software Inc., San Diego, CA, USA) with the ANOVA Student-*t* test. A *p*-value less than 0.5 was considered statistically significant. All the experiments were conducted in at least two independent setups, and the results were obtained in triplicate.

## 3. Results

### 3.1. In Vitro Release Profile of VEGF

**Figure 2:**
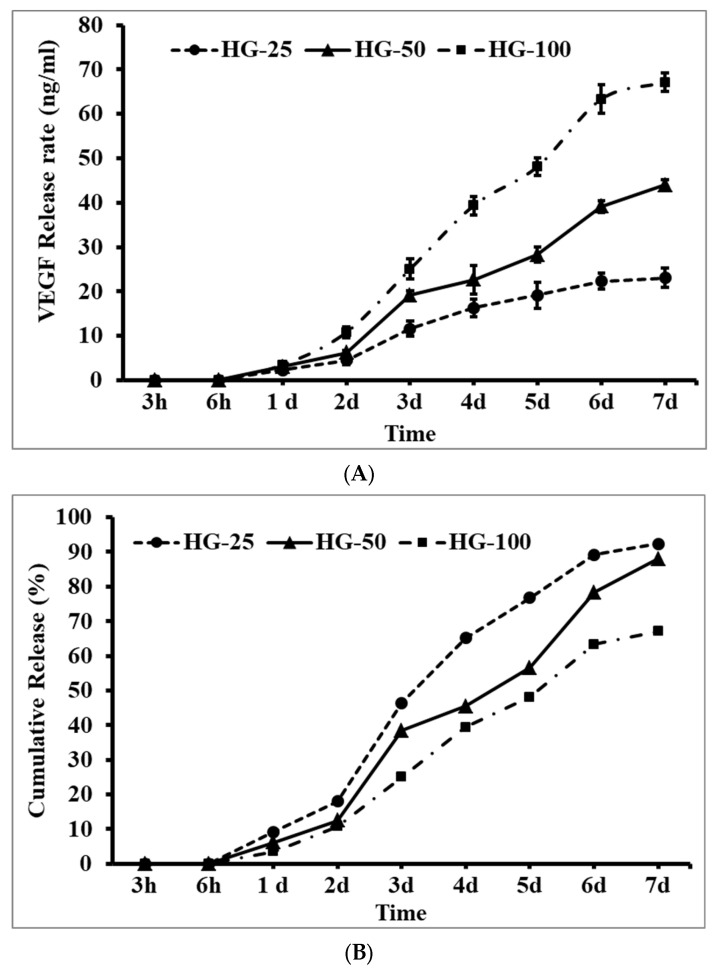
The release pattern of VEGF from hydrogels at different incubation times. (**A**) Actual release in ng/mL and (**B**) cumulative release in percentage. HG-25, HG-50, and HG-100—hydrogels with 25 ng/mL, 50 ng/mL, and 100 ng/mL of VEGF, respectively.

The amount of VEGF released from the HG over incubation was determined in order to link the biological activity of the HG in MSCs proliferation and differentiation. As shown in Figure 2, at the beginning (3 h and 6 h), a negligible or undetectable amount of VEGF was released from the HG; however, VEGF was released from the HG after 24 h incubation. In general, the release pattern of VEGF was increased with increasing incubation time from day 1 to day 7 in all three HGs. As expected, the release pattern of VEGF was higher in higher-concentrated VEGF-incorporated HGs (HG-100) than in lower concentrations (HG-50 and HG-25). Surprisingly, the VEGF content was abruptly released on day 3 in HG-25 and HG-50; on the other hand, this effect was observed on days 3 and 4 in HG-100. A significant release of VEGF was observed in HG-100 compared to lower-concentration HGs (HG-25 and HG-50), especially from day 3 to day 7 (*p* < 0.05). On day 7, the final percentage of VEGF release was approximately 92.4%, 87.96%, and 67.14% in HG-25, HG-50, and HG-100, respectively.

### 3.2. MSCs Proliferation

**Figure 3:**
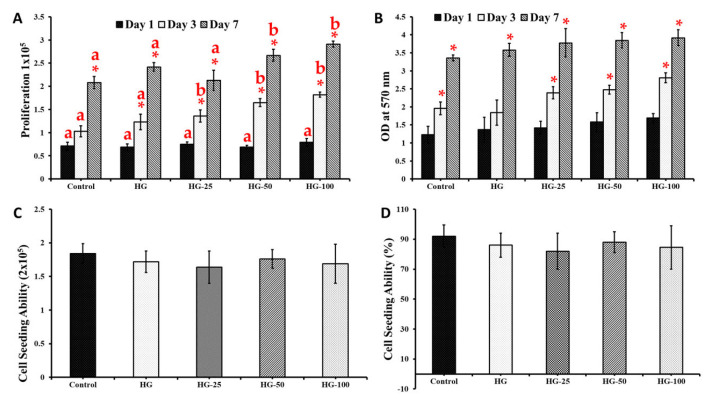
Proliferative (**A**), cytotoxic (**B**), and cell-seeding ability (**C**,**D**) of VEGF-loaded collagen–chitosan hydrogels. Control: cells without hydrogels, HG: hydrogels without VEGF, HG−25, HG−50, and HG−100: hydrogels with 25 ng/mL, 50 ng/mL, and 100 ng/mL of VEGF, respectively. * and different alphabets denote statistical significance, * vs. day 1 and different alphabets vs. control group, respectively, *p* < 0.05.

The effect of HGs and VEGF in MSC’s proliferation was carried out by cell counting assay. In general, the proliferative effect of cells steadily increased from day 1 to day 7 in all the groups (Figure 3A) and the proliferative effect was upregulated in HG-cultured cells compared to the control group (cells cultured without HG), especially on day 3 and 7 (*p* < 0.05). On the contrary, there was no significant difference observed between the control and HG groups on day 1, even with increasing VEGF concentration. Among the HG groups, the cell proliferative effect was upregulated in higher-concentration-loaded VEGF HGs, especially 50 and 100 ng/mL (HG-50 and HG-100) compared to the control HG (HG without VEGF) on days 3 and 7; however, no marginal difference was seen between the control HG and HG-25 (HG with 25 ng/mL of VEGF). The proliferative effect of HG-25 was slightly decreased compared to the control HG and the difference was not statistically significant. As expected, the proliferation rate of MSCs was greater in those cultured on high-concentration-loaded VEGF HGs (100 ng/mL) compared to 25- and 50 ng/mL-loaded HGs. In addition, the proliferation rate of MSCs was significantly increased by 100 ng/mL VEGF-loaded HG compared to the control group (*p* < 0.05).

### 3.3. Cytotoxicity of Hydrogels

The compatibility of HGs in MSCs was assessed by the MTT method and the results were presented in Figure 3B. The cells cultured on HGs showed no significant toxicity compared to the control. The formation of formazan crystals increased with respect to culture time (i.e., 1, 3, and 7 days) in all the groups. There was not much difference in the OD value at 570 nm between the control and HGs on day 3. However, the OD value was significantly increased in cells cultured on HGs loaded with VEGF (25, 50, and 100 ng/mL) (except the control HG without VEGF) compared to the control group (cells without HG) on day 3. On day 7, the OD value of the control (cells without HG) was similar to the control HG and HG-25 and lower than HG-50 and HG-100. The cytotoxic effect of the control HG and VEGF-loaded HG did not show any statistically significant differences. The above findings substantially supported the proliferative effect of HGs in MSCs (Figure 3A).

### 3.4. Cell Loading Ability

In order to estimate the total capacity of HGs in holding MSCs, the cells were seeded on HGs for 5 h and quantified by an automated cell counter. In general, the HG showed a similar cell loading ability as the control group and there was no significant difference among HGs (Figure 3C). Importantly, the cell loading ability of the HG was not influenced by the addition of VEFG. In terms of percentage, the control group showed a higher cell loading ability (92%) compared to HGs (84.5–88%); however, the data were not statistically significant between the control and HG groups (Figure 3D).

### 3.5. Morphological Analysis

**Figure 4:**
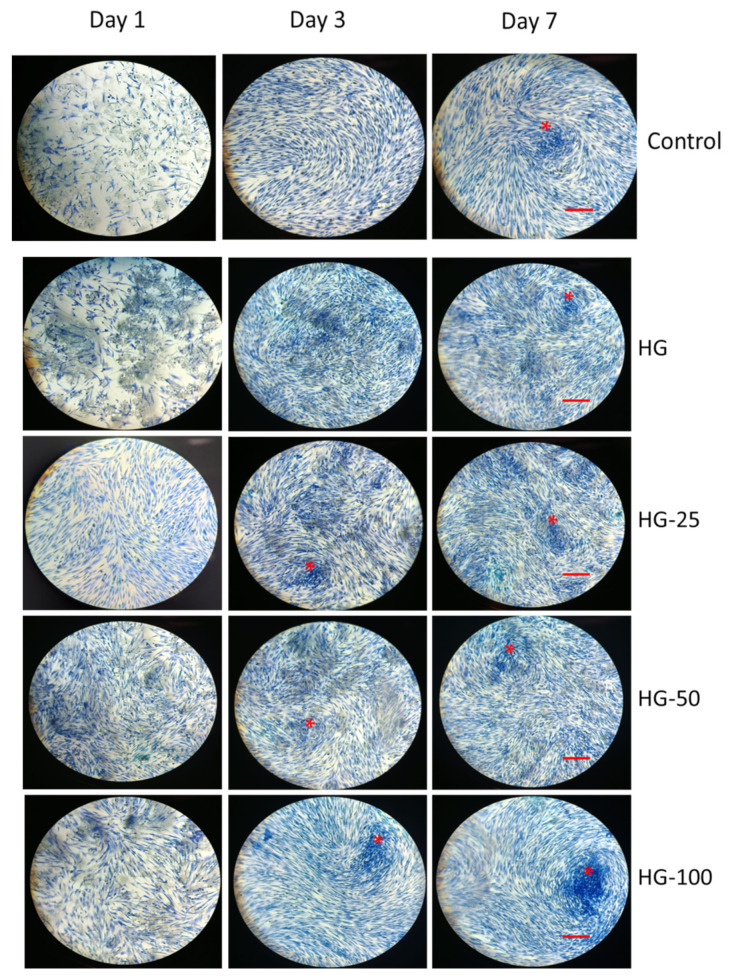
Morphological analysis of MSCs cultured on hydrogels by HE staining. Control—cells without hydrogels, HG—hydrogels without VEGF, HG-25, -50, and -100—hydrogels with 25 ng/mL, 50 ng/mL, and 100 ng/mL of VEGF, respectively. Scale bar 10×—100 μm. The asterisk indicates cellular aggregation and matrix deposition.

The cell morphology of MSCs cultured on HGs with and without VEGF was investigated by the H&E staining method. As observed in Figure 4, the H&E staining of MSCs was increased in 3- and 7-day cultures compared to day 1 in all the groups (control and HGs) (Figure 4). These results resemble the proliferative effect of MSCs cultured for 1, 3, and 7 days (Figure 3). The population of cells, in general, increased with increasing VEGF concentration of the HG, and an obvious difference was observed in the control and control HGs on day 1. It was clearly seen that the cells cultured on an HG with high VEGF concentrations contributed to a more fibrillary filament structure of MSCs compared to the control. On days 3 and 7, the cell population was quite high in those cultured on HGs with or without VEGF compared to the control. The increasing concentration of VEGF could increase the H&E staining more than the lower concentration. More importantly, the cells cultured on an HG with a high VEGF concentration for 7 days showed more cellular aggregation and matrix deposition compared to other groups as observed in Figure 4.

### 3.6. Cell Growth of Differentiated Osteogenic Cells

**Figure 5:**
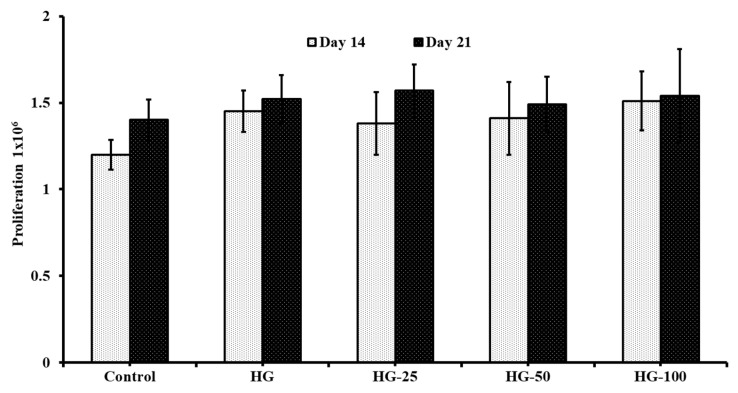
The cell growth of osteogenic cells differentiated from MSCs. Control—cells without hydrogels, HG—hydrogels without VEGF, HG-25, -50, and -100—hydrogels with 25 ng/mL, 50 ng/mL, and 100 ng/mL of VEGF, respectively.

Though the proliferation rate of undifferentiated MSCs increased by increasing the VEGF concentration, the cell growth of differentiated osteogenic cells did not improve with VEGF content in HGs (Figure 5) compared to control HGs on day 14 and day 21. However, the cells cultured on HGs had more cell growth (though it was statistically insignificant) in differentiated osteogenic cells compared to the control (without HGs) on day 14. Additionally, there was not much difference in the cell growth of differentiated osteogenic cells cultured on day 14 and day 21 in respective HGs.

### 3.7. Mineral Staining

**Figure 6:**
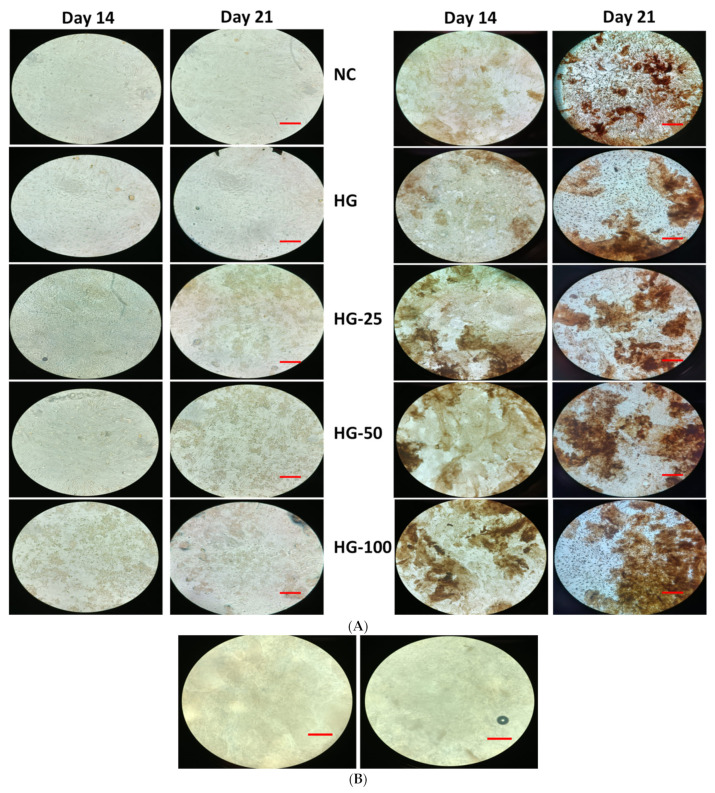
Von Kossa staining of osteogenic cells differentiated from MSC cultured on hydrogels for 14 and 21 days. Cells without osteogenic supplement (**Left**) and cells with osteogenic supplement (**Right**). (**A**) Cells cultured on hydrogels and stained, (**B**) only hydrogels with von Kossa stain without cells and supplement. NC—cells without hydrogels, HG—hydrogels without VEGF, HG-25, -50, and -100—hydrogels with 25 ng/mL, 50 ng/mL, and 100 ng/mL of VEGF, respectively. Scale bar 10×—100 μm.

**Figure 7:**
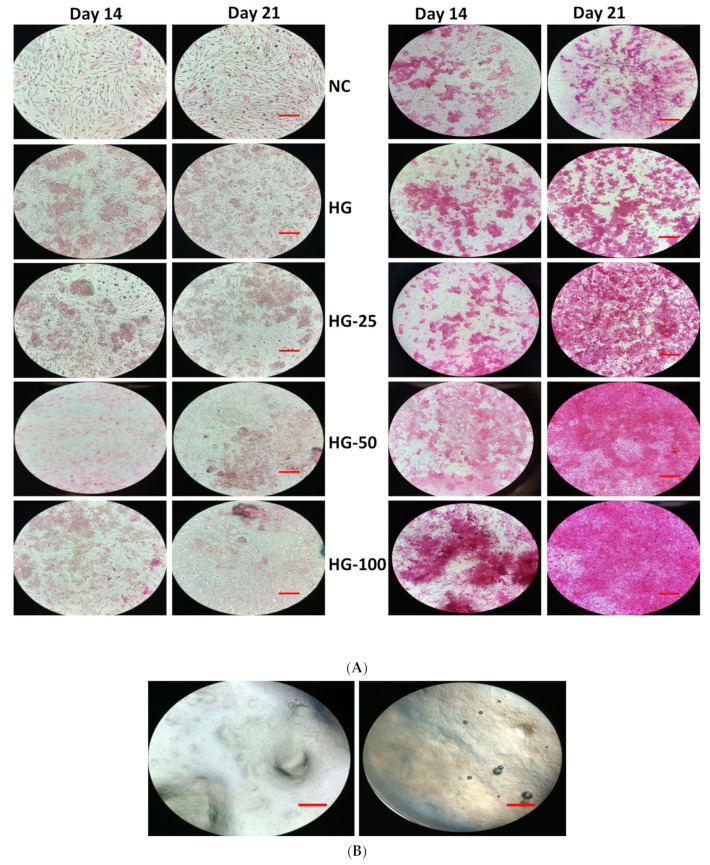
Alizarin red-S staining of osteogenic cells differentiated from MSC cultured on hydrogels for 14 and 21 days. Cells without osteogenic supplement (**Left**) and cells with osteogenic supplement (**Right**). (**A**) Cells cultured on hydrogels and stained, (**B**) only hydrogels with alizarin red S stain without cells and supplement. NC—cells without hydrogels, HG—hydrogels without VEGF, HG-25, -50, and -100—hydrogels with 25 ng/mL, 50 ng/mL, and 100 ng/mL of VEGF, respectively. Scale bar 10×—100 μm.

**Figure 8:**
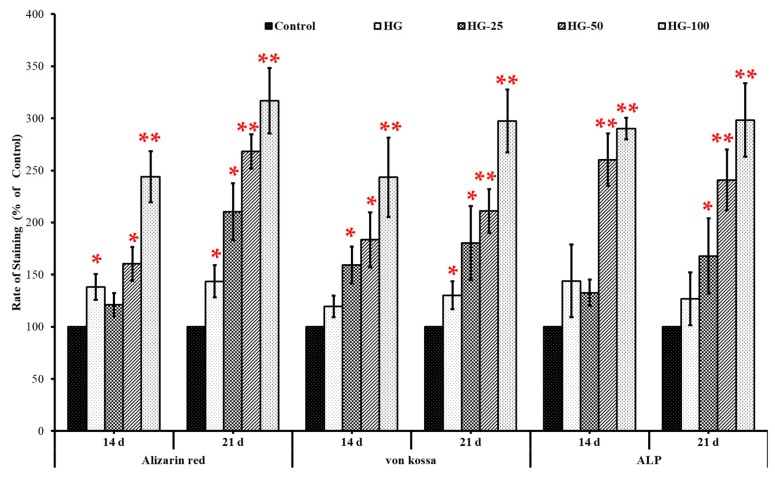
Quantification of the stained area of differentiated bone marrow mesenchymal stem cells cultured on hydrogels. The percentage of stained area in osteogenic cells was quantified using ImageJ software (Version 1.52n). Control—cells without hydrogel, HG—hydrogels without VEGF, HG-25, HG-50, and HG-100—hydrogels with 25 ng/mL, 50 ng/mL, and 100 ng/mL of VEGF, respectively. ALP-alkaline phosphatase. * *p* < 0.05 vs. control, ** *p* < 0.01 vs. control.

The mineralization ability of MSCs with and without HGs was substantially confirmed by von Kossa and alizarin red S stains. The cells cultured on HGs without an osteogenic culture medium or osteogenic supplements did not show any positive staining for von Kossa and alizarin red, even with increasing VEGF concentration in HGs (Figure 6A and Figure 7A). Additionally, there was no positive staining observed in HGs treated with von Kossa and alizarin red S stains alone without MSCs (blank) (Figure 6B and Figure 7B). As expected, the control cells (without HG) showed positive staining for von Kossa and alizarin red S stains on days 14 and 21, and the rate of mineral formation was increased with increasing culture duration, showing more positive stains on day 21 culture compared to day 14. There was not much difference in the staining pattern of control cells and control HG cells on day 14; on the contrary, the cells produced more minerals than those cultured on control HGs compared to control cells on day 21 (Figure 8). The rate of von Kossa and alizarin red S staining was higher in cells cultured on HGs with a VEGF content compared to control HGs and control cells on days 14 and 21. Specifically, the cells produce more apatite layers when cultured on an HG with a higher concentration (100 ng/mL) compared to other VEGF-loaded HGs (25 and 50 ng/mL) on day 21.

### 3.8. Alkaline Phosphatase Staining

**Figure 9:**
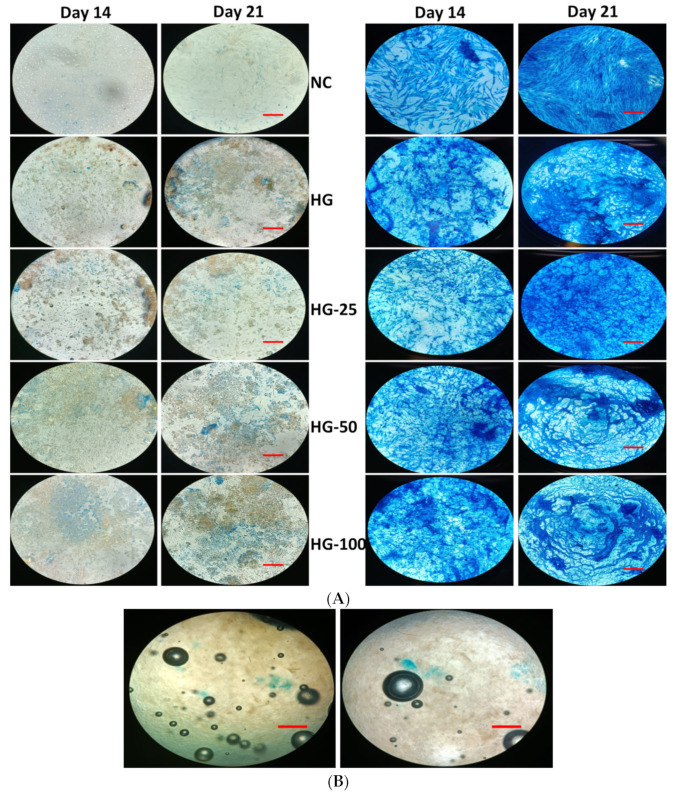
Alkaline phosphatase staining of osteogenic cells differentiated from MSC cultured on hydrogels for 14 and 21 days. Cells without osteogenic supplement (**Left**) and cells with osteogenic supplement (**Right**). (**A**) Cells cultured on hydrogels and stained, (**B**) only hydrogels with ALP stain without cells and supplement. NC—cells without hydrogels, HG—hydrogels without VEGF, HG-25, -50, and -100—hydrogels with 25 ng/mL, 50 ng/mL, and 100 ng/mL of VEGF, respectively. Scale bar 10×—100 μm.

Unlike mineral staining, the cells cultured for 14 and 21 days without osteogenic culture medium and osteogenic supplements showed slight positive staining for ALP and the rate of staining was increased with increasing culture time from 14 to 21 days (Figure 9). Surprisingly, the cells expressed more ALP level in VEGF-loaded HGs compared to control and expression level was more pronounced in cells cultured on high VEGF-loaded HGs compared to other VEGF-loaded HGs and control HGs.

Similar to mineral staining, the cells with an osteogenic culture medium and osteogenic supplements expressed more positive staining for ALP both on days 14 and 21 and the level of expression was increased with increasing culture time. The network formation of cells on HGs is clearly seen by ALP staining in Figure 9. On day 14, the amount of ALP staining was higher in cells cultured on a control HG compared to control cells and at the same time, the level of ALP was expressed more in VEGF-loaded HGs than in control HGs on days 14 and 21. Among the HGs, the VEGF-loaded HGs had more ALP staining than control HGs and the effect was more pronounced in a higher concentration of VEGF-loaded HGs compared to other VEGF-loaded HGs. The blank HGs treated without cells, an osteogenic culture medium, or osteogenic supplements did not show any positive staining for ALP. These observations further supported the mineral staining profile of HG-cultured cells (Figure 6 and Figure 7).

## 4. Discussion

Empirical evidence claims that MSCs are smart candidates for stem cell therapy in regenerative tissue engineering thanks to their regenerative and multipotent properties, which permit them to be used in various clinical contexts [50,51]. In the present study, we evaluated the proliferative and osteogenic stimulatory properties of HGs fabricated with different concentrations of VEGF in addition to the release profile. Our results showed that all the HGs showed a better drug-releasing ability of VEGF over the incubation time and higher VEGF drug release was observed with a higher concentration of VEGF-loaded HGs. The drug release profile further demonstrates that the HG could start releasing quantifiable VEGF after 6 h incubation and a significant release of VEGF by HGs was achieved on day 3 onwards, which is contradictory to the previous studies [52,53,54]. This could be due to the influence of the polymer’s nature, such as the type, composition, and chemical bonding of the polymers, on the drug-releasing behavior of hydrogels. The higher release rate of VEGF in HG-100 could justify the better biological activity of HG-100 compared to HG-25 and HG-50. For instance, the proliferative effect of MSCs was upregulated by culturing cells on HGs as well as increasing VEGF contents compared to the control group. The higher proliferative effect of MSCs cultured on VEGF-loaded HGs was due to the efficient liberation of VEGF from HG into the culture medium, which stimulated the proliferative genes in MSCs and thus increased the cells’ numbers. To prove this hypothesis, previously Yun et al., investigated the molecular mechanism of VEGF in the proliferation of stem cells and found that VEGF increased cell proliferation through modulating PKC, PI3K/Akt, and MAPK signaling pathways [55]. The present study result claims the suitability and appropriateness of this HG system in culturing stem cells. Our study also confirmed that the cells cultured on HGs with or without VEGF did not show any cytotoxic effect, even with a higher concentration of VEGF, which further proves the biocompatibility of HGs. Similar to the present study, the proliferation of MSCs was improved by the VEGF-loaded HGs such as the fibrin and hyaluronic acid HG [56,57] and chitosan/PVA HG [58].

The cell loading ability of HGs proved the excellent binding capacity of HGs with MSCs as similar to cell culture plates. However, the cell loading ability of HGs was not promoted by adding VEGF, since no statistical difference was observed between control HGs and VEGF-loaded HGs. However, the cells’ morphological features were altered by VEGF contents in the HG; for instance, the cells had more elongated fibrillary filaments than those cultured with VEGF-loaded HGs compared to the control. To support this finding, a previous study suggested that VEGF-loaded poly(ethylene glycol) HGs served as an excellent platform to stimulate endothelial cell migration, cell–cell communication, and proliferation [59]. VEGF is recognized as a potent angiogenic factor that upregulates the covalent interaction between cells and the adhesive peptide RGDS of collagen monomers [60]. The actual reason behind the stimulatory effect of VEGF-loaded collagen–chitosan HGs is that the HG system liberated a soluble form of VEGF during the culture of MSCs, which potentially triggers the proliferation of MSCs. The above statement was justified by the drug-releasing pattern of VEGF from hydrogels on days 3–7 (Figure 2). Previous reports also stated that the proliferation of MSCs was mediated by the interaction of cell membrane integrins such as integrin αβ with collagen [15] and VEGF [61,62].

In the present study, the cell growth of differentiated osteogenic cells did not alter in 14- and 21-day cultures in all the treatment groups and control, which was contradictory to the previous reports [49,63,64]. We speculate that this could be due to the longer culture duration of MSCs on HGs since the cells tend to confluence in 7 to 10 days and then reach a stationary phase where the cells’ growth was limited.

The results of histological mineral staining with von Kossa, alizarin red S, and ALP show that the HGs treated without MSC cells did not show any positive staining, which provides concrete evidence that the HG did not react with any of the histological stains used in this study. Interestingly, the cells cultured on HGs with VEGF had more mineral and ALP staining which directly shows that the VEGF supports the osteogenic differentiation of MSCs cultured on HGs in the presence of osteogenic supplements. However, the cells cultured on VEGF-loaded HGs without an osteogenic culture medium and osteogenic supplement failed to stain positively for three stains (von Kossa, alizarin red S, and ALP), even in cells cultured in higher concentrations (100 ng/mL). Additionally, the collagen–chitosan HG alone failed to induce osteogenic differentiation of MSCs without an osteogenic supplement. All these data confirmed that the HGs and VEGF could not induce the osteogenic differentiation of MSCs unless they were supplemented with osteogenic supplements. However, the osteogenic differentiation ability of MSCs cultured with an osteogenic culture medium and supplements was upregulated by VEGF-loaded HGs and control HGs compared to control cells (without HGs). Surprisingly, the osteogenic supportive effect of HGs was increased by increasing the VEGF content in HGs, which proved the osteogenic stimulatory effect of VEGF. Previous studies also proved the osteogenic stimulatory effect of HGs fabricated with nano-hydroxyapatite/collagen [6,65], chitosan/collagen [17,48], and hyaluronic acid/collagen [66]. Similarly, the osteogenic stimulatory effect of VEGF loaded in HGs was also demonstrated in several reports [37,54,67,68]. Several pieces of empirical evidence elucidated the actual signaling mechanism of VEGF in the osteogenesis of MSCs. Based on the literature, VEGF enhances the epithelization and deposition of collagen and minerals by stimulating the expression of BMP-2, osteocalcin, and ERK1/2 MAP kinase [25,69]. Overall, the control HGs and VEGF-loaded HGs could support the osteogenic differentiation of MSC cells in the presence of osteogenic inducers, but the osteogenesis of MSCs could not be induced by HGs alone without stimulators.

## 5. Conclusions

In the present study, the proliferative, cell-seeding, and osteogenic stimulatory properties of HGs fabricated with or without VEGF were investigated. Our results showed that the proliferative effect of MSCs was accelerated by composite collagen and chitosan HGs loaded with different concentrations of VEGF and the effect was more pronounced with increasing VEGF contents compared to the control group. Interestingly, the cells cultured on different HGs with or without VEGF did not show any cytotoxic effects, which further proved the biocompatibility of HGs in culturing MSCs. In addition, the osteogenic stimulatory effect of MSCs in the presence of osteogenic supplements was accelerated by HGs with or without VEGF; however, the stimulatory effect was more pronounced in cells cultured on higher VEGF-loaded HGs. In contrast, either HGs or VEGF-loaded HGs alone did not induce the osteogenic differentiation of MSCs unless supplemented with osteogenic inducers. On the other hand, HGs loaded with VEGF accelerated the deposition of mineral and ALP levels in osteogenic cells compared to the control in the presence of osteogenic inducers. This result concluded that the HGs loaded with VEGF could improve the osteogenesis of MSCs in the presence of an inducer, but not in its absence. Overall, the fabricated HGs with VEGF could upregulate the proliferation and mineralization of differentiated MSCs and thereby support the osteogenesis of mesenchymal stem cells. However, more studies are essential to prove the osteogenic supporting effect of these HGs in order to investigate the molecular signaling mechanism by mRNA and protein expressions.

## Data Availability

Not applicable.

## Supplementary Materials

The following supporting information can be downloaded at: https://www.mdpi.com/article/10.3390/pharmaceutics15041297/s1. Figure S1: The cell culture plate coated with collagen–chitosan hydrogels before mesenchymal stem cell seeding.
