# Supplementary material for: Proliferative and Osteogenic Supportive Effect of VEGF-Loaded Collagen-Chitosan Hydrogel System in Bone Marrow Derived Mesenchymal Stem Cells
Source: Pharmaceutics. 2023 Apr 20;15(4):1297. doi: 10.3390/pharmaceutics15041297 (PMC10143960; doi:10.3390/pharmaceutics15041297)
Supplement: Supplementary file 1 [file pharmaceutics-15-01297-s001.zip › pharmaceutics-2296791-supplementary.pdf]

Article

Proliferative and Osteogenic Supportive Effect of VEGF-loaded Collagen-Chitosan Hydrogel System in Bone Marrow derived Mesenchymal Stem Cells

Jeevithan Elango [1,2,3]

[1] Department of Biomaterials Engineering, Faculty of Health Sciences, UCAM-Universidad Católica San Antonio de Murcia, Campus de los Jerónimos 135, Guadalupe, 30107 Murcia, Spain; srijeevithan@gmail.com or jelango@ucam.edu; Tel.: +34-6-0359-7596

[2] Center of Molecular Medicine and Diagnostics (COMManD), Department of Biochemistry, Saveetha Dental College and Hospitals, Saveetha Institute of Medical and Technical Sciences, Saveetha University, Chennai 600077, India

[3] Department of Marine Biopharmacology, College of Food Science and Technology, Shanghai Ocean University, Shanghai 201306, China

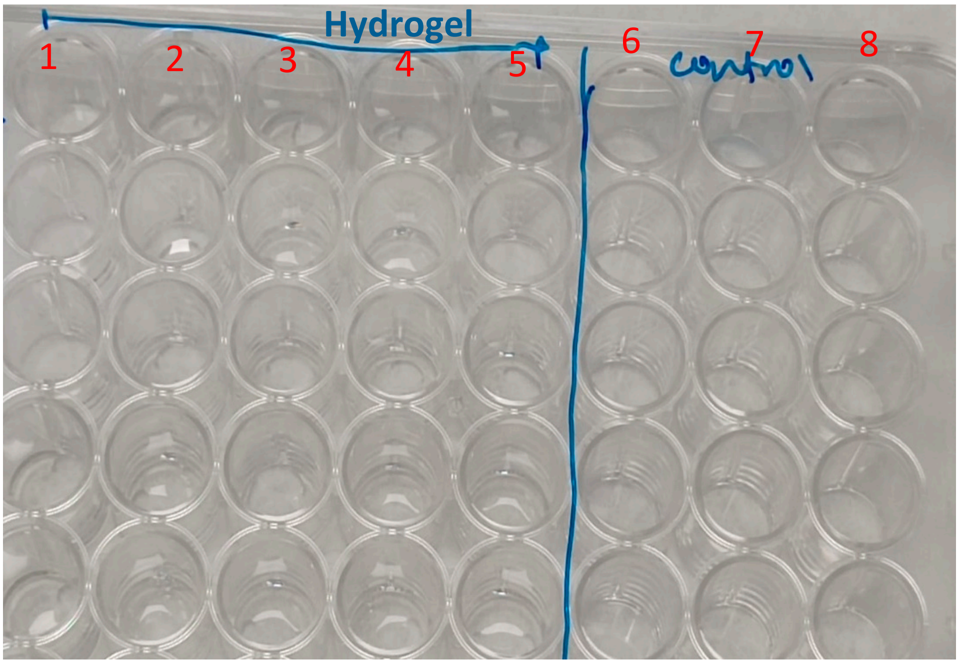

Supplementary Figure S1. The cell culture plate coated with collagen-chitosan hydrogels before mesenchymal stem cell seeding. 1:-PBS, 2:-HG, 3:-HG-25, 4:-HG-50, 5:- HG-100 and 6-8:- control groups.
